# Consumers’ Health-Related Motive Orientations and Reactions to Claims about Dietary Calcium

**DOI:** 10.3390/nu5010082

**Published:** 2013-01-10

**Authors:** Christine Hoefkens, Wim Verbeke

**Affiliations:** Department of Agricultural Economics, Faculty of Bioscience Engineering, Ghent University, Coupure Links 653, B-9000 Ghent, Belgium; E-Mail: Wim.Verbeke@ugent.be

**Keywords:** nutrition and health claims, calcium, enriched fruit juice, consumer, health-related motive orientations, motives

## Abstract

Health claims may contribute to better informed and healthier food choices and to improved industrial competitiveness by marketing foods that support healthier lifestyles in line with consumer preferences. With the more stringent European Union regulation of nutrition and health claims, insights into consumers’ health-related goal patterns and their reactions towards such claims are needed to influence the content of lawful claims. This study investigated how consumers’ explicit and implicit health-related motive orientations (HRMOs) together with the type of calcium-claim (nutrition claim, health claim and reduction of disease risk claim) influence perceived credibility and purchasing intention of calcium-enriched fruit juice. Data were collected in April 2006 through a consumer survey with 341 Belgian adults. The findings indicate that stronger implicit HRMOs (*i.e.*, indirect benefits of calcium for personal health) are associated with higher perceived credibility, which is not (yet) translated into a higher purchasing intention. Consumers’ explicit HRMOs, which refer to direct benefits or physiological functions of calcium in the body—as legally permitted in current calcium-claims in the EU—do not associate with reactions to the claims. Independently of consumers’ HRMOs, the claim type significantly affects the perceived credibility and purchasing intention of the product. Implications for nutrition policy makers and food industries are discussed.

## 1. Introduction

Although not legally defined by the European Union (EU), functional foods are generally considered as foods in a regular diet that offer a particular health benefit beyond their regular nutritional value [1]. Examples of functional foods include foods enriched with specific minerals, vitamins, fatty acids, dietary fiber, phytochemicals or other antioxidants. Functional foods offer the potential to improve public health when consumed as part of a balanced diet and healthy lifestyle [2]. The potential health benefits from functional foods are mainly communicated to consumers by means of food claims on product packages. From a consumer point of view, food claims are important information cues that can guide their food choices, enable them to make better informed and healthier food choices and, if appropriately regulated, protect them against unsubstantiated or misleading statements about foods [3,4]. At the same time, food claims are used by the food industry to better differentiate their products in the market and, hence, to build and maintain health-oriented competitive advantages [5,6,7]. Moreover, food claims allow food companies to make their corporate social responsibility visible to the public [8]. 

According to the Regulation (EC) 1924/2006 on nutrition and health claims made on foods (the Regulation) that came into force in July 2007, two types of claims may be used on food products: nutrition claims and health claims [9]. Nutrition claims refer to a food’s particular beneficial nutritional properties, because of specific nutrients it contains in reduced or increased proportion or does not contain. Whereas health claims state the relation between the nutrients and health. Reduction of disease risk claims are a specific category of health claims that link the food with a specific disease. Food claims in general and reduction of disease risk claims specifically are strictly regulated under the Regulation: claims should be based on sound scientific evidence and formulated in a way that an average consumer can understand the beneficial effects of the food. Consequently, foods bearing claims that could mislead consumers, because they are not supported by the evidence or cannot be understood, are prohibited. 

Effective use of nutrition information, including food claims, is a question of not only liking and understanding, but also motivation [10]. While correct understanding of food claims may be sufficient to foster informed food choices, actual healthy choices will largely depend on whether consumers find the claimed health effect sufficiently relevant for themselves in the broader context of food choice. Although consumer reactions to food claims have been studied widely [11,12], very few studies have attempted to measure the understanding of claims [13] and motivational factors underpinning consumers’ reactions to claims [14,15]. The latter will be the focus of this study, while understanding of health claims will be one of the main research questions of CLYMBOL (Role of health-related claims and symbols in consumer behavior), which is a recently funded project within FP7 of the European Commission (FP7-KBBE-6-2012-311963). 

Given that health is an important motivator of consumers’ food choice behavior [16], consumers’ health-related motive orientations (HRMOs) in association with consumers’ reactions to functional foods will be studied. HRMOs are defined as the psychological meanings that people attribute to health and that motivate health behavior [17,18]. Two independent motivational systems are distinguished, *i.e.*, an emotion-driven implicit system of non-conscious motives *versus* a cognition-based explicit system constituted of more conscious goals and values [19]. It is expected that people have different health-oriented goals and needs, which may explain differences in health behavior and, thus, differences in the potential effectiveness between food claims that mainly refer to the explicit HRMOs as a result of the provisions of the current EU regulation. Despite the fact that the content of health claims in particular is restricted by law, food companies still have some degree of freedom, including the specific way in which the health claim is communicated [20]. A better understanding of consumers’ explicit and implicit HRMOs and their association with consumers’ reactions towards functional foods will therefore contribute to the development of potentially more effective nutrition and health claims and communications.

The objective of the present study was to investigate how consumers’ explicit and implicit HRMOs together with the type of calcium-claim (nutrition claim, health claim and reduction of disease risk claim) would influence their reactions towards calcium-enriched fruit juice. Consumers’ reactions were assessed as perceived credibility and intention to buy the product, while accounting for differences in product familiarity, socio-demographic and attitudinal characteristics. Calcium-enriched fruit juice was chosen as the object of study owing to: (1) its high consumption frequency [21] and (2) its specific nature as functional food, namely a non-natural type of enrichment (*i.e.*, fruit juice is not a typical natural source of dietary calcium) [22] in a carrier product that is, despite its apparent association with healthy food (its fruit-based origin) [23], increasingly contested for its high energy and sugar content [24]. The findings of this study are valuable for improving consumer-oriented positioning and marketing of functional foods.

## 2. Methods

### 2.1. Study Design and Population

Data were collected in April 2006 through a cross-sectional quantitative survey with 341 Belgian consumers. Participants were volunteers who were recruited through random walk sampling [25] in ten randomly selected Belgian cities and villages. Interviewers were given a random start address and walked from house to house following a predefined set of rules to proceed to the next contact. This sampling approach is a valuable alternative to probability sampling in cases where a sampling frame is not available [26]. Upon prospect’s agreement to participate and after receiving their informed consent, the questionnaire was distributed, self-administered by the participants and collected a couple of hours later. Data were coded and stored in a non-identifiable format, and they were processed fully anonymously.

The mean age of the participants was 37.4 years (SD = 14.3) and 56% were female. Compared to census data for Belgium [27], there was a slight overrepresentation of the younger age groups and female participants. With respect to living environment, 31% were living in urban areas, 41% in mid-sized towns and 28% in rural areas. About 42% of the participants indicated to have children below the age of 18 living in their households, while 13% reported to have children below the age of 12 living in their households. The distributions for living environment and presence of children matched closely with population census data for Belgium. The sample was characterized by an overrepresentation of higher educated adults. Two-thirds of the participants reported to have received education beyond the age of 18 years. Owing to our interest in studying the effect of HRMOs in relation to product familiarity (non-users *versus* different user categories), the sample was not restricted to consumers of calcium-enriched fruit juice alone. However, people who claimed to be not aware of the product concept (although very few) were excluded during participant recruitment.

### 2.2. Measures

#### 2.2.1. Dependent Variables

***Perceived credibility***. The first outcome variable was the perceived credibility of calcium-enriched fruit juice with a claim. Participants were asked to evaluate the credibility of each of the three fruit juice claim combinations (nutrition claim, health claim and reduction of diseases risk claim). The response scales were seven-point interval scales ranging from “Not credible at all” (=1) to “Very much credible” (=7). In order to avoid presentation order bias, the order of claims was rotated across participants.

***Buying intention***. The second outcome variable was consumers’ intention to buy the calcium-enriched fruit juice, which was similarly assessed for the three claim types on a seven-point interval scale ranging from “No intention to buy at all” (=1) to “Very likely to buy” (=7). 

#### 2.2.2. Independent Variables: Main and Interaction Effects

***Claim type***. Three types of food claims related to calcium-enriched fruit juice were investigated: (1) a nutrition claim, *i.e.*, “Fruit juice enriched with calcium”, (2) a health claim, *i.e.*, “Fruit juice enriched with calcium can strengthen bones”, (3) reduction of disease risk claims, *i.e.*, “Fruit juice enriched with calcium reduces risk in the development of osteoporosis”.

***Health-related motive orientations (HRMOs)***. Participants’ HRMOs were assessed by means of a selected number of items from the 45-item HRMO-scale developed by Geeroms *et al.* [17,18]. Only items that were related to the function of calcium in the human body were selected for analysis. This resulted in four items for explicit HRMO, with its focus on direct benefits of calcium for personal health: “For me, health is mainly about... (1) keeping the body in a good condition (fitness, jogging, and aerobics); (2) having the energy to do things I want to do; (3) having no physical health problems; (4) reducing physical health risks with regard to heart, lungs, liver”. The three other selected items assessed HRMO that are implied rather than explicit by focusing on indirect benefits of calcium for personal health: “Because of health problems, it would be (extremely) bad not to be able anymore to… (1) help others; (2) feel secure in life; (3) practice sports”. All HRMOs were measured on a seven-point Likert scale ranging from “Totally disagree” (=1) to “Totally agree” (=7). For both the explicit and implicit HRMOs, three subgroups (low, medium, high) were defined based on tertiles of the respective construct. 

#### 2.2.3. Independent Variables: Covariates

***Socio-demographic characteristics***. Because men and younger age groups were previously found to perceive foods with claims more credible than women and older consumers [28], gender and age were included as covariates in the analysis. In general, mixed results were found in previous studies regarding gender and age effects on consumer reactions to functional foods and claims [29,30,31,32,33,34].

***Product familiarity***. Familiarity with the product, health claim and/or functional ingredient emerges as one of the most important determinants of consumers’ acceptance of functional foods or foods with health claims [12]. Consumer’s familiarity or past experience with calcium-enriched fruit juice was assessed using a six-point consumption frequency scale, ranging from “Never” (score 0) to “Daily” (score 5). For the descriptive analyses, participants were divided in user groups (non-users, light, medium and heavy users) on the basis of tertiles of their consumption frequency score (non-users excluded).

***General attitude towards functional foods***. Beside consumers’ socio-demographics and their product familiarity, also attitudes towards functional foods were reported to influence their reactions to functional foods and claims [28,34]. Therefore, this background attitude was measured and included as a covariate in the analysis using four items on seven-point semantic differential scales, including “Unattractive/Attractive”, “Not interesting/Interesting”, “Unimportant/Important” and “Negative/Positive” [35]. Cronbach’s alpha for this four-item measure was 0.89, indicating very good internal consistency reliability [36]. Therefore, the scores were merged into one general attitude score. Similarly, as for product familiarity, participants were classified on the basis of tertiles (negative, neutral and positive attitudes) according to their general attitude towards functional foods.

### 2.3. Statistical Analysis

Descriptive analyses were used to describe the dependent variables (perceived credibility, buying intention) by a combination of HRMOs and one other independent variable (claim type, gender, age group, product familiarity, general attitude towards functional foods). Results are presented as means (M) and standard deviations (SD). Differences were analyzed through one-way ANOVA F-tests with Bonferroni post-hoc estimation (in case of parametric data distributions) and Kruskal-Wallis tests (in case of non-parametric data distributions). 

A linear mixed model was performed to simultaneously investigate the main and interaction effects of explicit and implicit HRMOs and claim type on the perceived credibility of calcium-claims on fruit juice (Model 1) and buying intentions of fruit juice with calcium-claims (Model 2), while accounting for differences in product familiarity, socio-demographic and attitudinal characteristics. Explicit and implicit HRMOs were specified as between-subjects factors, and claim type was included as within-subjects factor in the models. 

Statistical analyses were performed using the statistical software program IBM SPSS 20.0 (SPSS Inc., Chicago, IL, USA) [37]. Statistical significance was set at α = 0.05. All statistical tests were 2-sided.

## 3. Results

### 3.1. Associations between Claim Type and HRMOs Linked to Calcium

The perceived credibility and buying intention of calcium-enriched fruit juice bearing a reduction of disease risk calcium-claim, were the lowest among participants with low explicit and low to medium implicit calcium-related HRMOs compared to the other combinations of claim type and intensity of HRMO (Table 1). The health claim was assessed as the highest in product credibility and buying intention for the subgroup of participants with the strongest explicit HRMOs. Participants with high implicit HRMOs rated the credibility and buying intention of calcium-enriched fruit juice with the nutrition or health claim the highest. A similar pattern was observed for the association between claim type and the intensity of HRMO regardless of the outcome variable (perceived credibility, buying intention), without taking differences in product familiarity, socio-demographic and attitudinal characteristics into account. This can be explained by the significant correlation found between the perceived credibility and buying intention of a product (*r* = 0.74, *p* < 0.001). This means that the more credible the claim is perceived, the stronger the intention to buy the product. 

### 3.2. Associations between Covariates and HRMOs Linked to Calcium

Differences in perceived credibility of calcium-claims were found according to participants’ implicit HRMOs and their gender, though not by explicit HRMOs (Table 2). Men with high implicit HRMOs perceived the calcium-enriched fruit juice with claims significantly more credible than men and women with low to medium implicit HRMOs. Intention to buy calcium-enriched fruit juice was the lowest among men with low explicit HRMOs and the highest for women with high implicit HRMOs (Table 3). 

There is no clear relation between participants’ explicit and implicit HRMOs by age and the perceived credibility and purchasing intention of calcium-enriched fruit juice with claims (Table 2 and Table 3). The two age groups with the largest contrast in perceived credibility were the age group of 26–40 years and above 50 age group, while for purchasing intention the youngest age group (18–25 years) differed significantly from the above 50 age group regardless of the intensity of HRMOs. In both cases the age group of 50+ showed the lowest perceived credibility and purchasing intention of calcium-enriched fruit juice bearing claims.

Participants, who were more familiar with the calcium-enriched fruit juice, perceived the calcium-claims as more credible (Table 2) and expressed higher intentions to buy the product in the future (Table 3) regardless of the intensity of their HRMOs. Perceived credibility and intention to buy were found to be the highest among heavy users and the lowest among non-users of calcium-enriched fruit juice. 

No clear association was found between participants’ HRMOs by their attitude towards functional foods and the perceived credibility and purchasing intention of calcium-enriched fruit juice with claims (Table 2 and Table 3). Regardless of the intensity of participants’ HRMOs, the perceived credibility and purchasing intention of the product were higher, with more favorable attitudes towards functional foods. 

**Table 1:** Perceived credibility of and intention to buy fruit juice with calcium claim by claim type and health-related motive orientations (HRMO) [Mean (Standard Deviation)].

HRMO		Perceived credibility			Intention to buy		
		NC	HC	RR	NC	HC	RR
Explicit	Low	3.71 (1.75) ^a,b^	3.78 (1.72) ^a,b^	3.31 (1.50) ^a^	3.44 (1.80) ^a,b,c^	3.37 (1.82) ^a,b^	3.07 (1.72) ^a^
	Medium	4.03 (1.71) ^a,b^	3.81 (1.60) ^a,b^	3.49 (1.54) ^a,b^	3.93 (2.09) ^c,d^	3.82 (1.96) ^b,d^	3.54 (1.86) ^a,b,c^
	High	3.97 (1.85) ^a,b^	4.09 (1.82) ^b^	3.69 (1.81) ^a,b^	3.87 (2.17) ^b,d^	4.04 (2.17) ^d^	3.76 (2.20) ^b,d^
Implicit	Low	3.85 (1.83) ^a,b^	3.80 (1.72) ^a,b^	3.28 (1.47) ^a^	3.68 (2.04) ^a,b,c,d^	3.60 (1.93) ^a,b,c^	3.15 (1.79) ^a^
	Medium	3.56 (1.80) ^a,b^	3.61 (1.66) ^a,b^	3.34 (1.63) ^a^	3.45 (2.12) ^a,b^	3.44 (2.08) ^a,b^	3.22 (2.03) ^a^
	High	4.18 (1.69) ^b^	4.15 (1.73) ^b^	3.79 (1.73) ^a,b^	4.03 (2.00) ^c,d^	4.11 (2.00) ^d^	3.91 (2.00) ^b,c,d^

The letters ^a–d^ indicate significantly different means on a seven-point interval scales ranging from “Not at all” (=1) to “Very much” (Perceived credibility) (=7) or “Very likely” (Buying intention); Abbreviations: NC = nutrition claim; HC = health claim; RR = reduction of disease risk claim.

**Table 2:** Perceived credibility of calcium claim on fruit juice [Mean (Standard Deviation)] by explicit and implicit HRMO and covariates.

		Explicit HRMO			Implicit HRMO		
		Low	Medium	High	Low	Medium	High
Gender	Male	3.54 (1.69) ^a^	3.78 (1.55) ^a^	4.08 (1.78) ^a^	3.53 (1.76) ^a,b^	3.73 (1.70) ^a,b,c^	4.10 (1.58) ^d^
	Female	3.66 (1.65) ^a^	3.77 (1.69) ^a^	3.78 (1.84) ^a^	3.76 (1.63) ^b,c^	3.36 (1.68) ^a^	3.98 (1.81) ^c,d^
Age group	18–25	3.74 (1.64) ^a,b,c,d^	4.08 (1.31) ^d,e^	4.00 (1.78) ^c,d,e^	3.99 (1.43) ^d,e^	3.80 (1.74) ^b,c,d,e^	3.98 (1.59) ^d,e^
	26–40	3.57 (1.25) ^a,b,c,d^	3.83 (1.64) ^c,d,e^	4.19 (1.62) ^e^	3.60 (1.85) ^b,c,d^	4.06 (1.39) ^c,d,e^	4.17 (1.49) ^e^
	41–50	3.95 (1.85) ^b,c,d,e^	3.50 (1.74) ^a,b,c^	3.86 (2.08) ^a,b,c,d,e^	3.64 (1.85) ^b,c,d,e^	3.41 (1.87) ^a,b,c^	4.07 (1.96) ^d,e^
	50+	3.25 (1.71) ^a^	3.50 (1.87) ^a,b,c^	3.32 (1.87) ^a,b^	3.36 (1.70) ^a,b^	2.83 (1.46) ^a^	3.89 (2.13) ^b,c,d,e^
Product familiarity	Non-user	2.84 (1.56) ^a^	3.14 (1.66) ^a^	2.93 (1.71) ^a^	2.73 (1.44) ^a^	2.88 (1.58) ^a,b^	3.25 (1.83) ^b,c^
	Light user	4.14 (1.58) ^b,c^	3.82 (1.52) ^b^	4.13 (1.64) ^b,c^	4.08 (1.51) ^e,f^	3.55 (1.77) ^c,d^	4.33 (1.40) ^f^
	Medium user	4.33 (1.11) ^b,c^	4.47 (1.13) ^c,d^	4.09 (1.33) ^b,c^	4.90 (1.03) ^g^	4.61 (1.42) ^f,g^	3.81 (1.00) ^d,e^
	Heavy user	4.24 (1.66) ^b,c^	4.55 (1.52) ^c,d^	4.93 (1.75) ^d^	4.49 (1.85) ^f,g^	4.36 (1.25) ^f,g^	4.84 (1.72) ^g^
General attitude towards functional foods	Negative	3.15 (1.75) ^a^	3.38 (1.65) ^a,b^	3.42 (2.05) ^a,b,c^	3.28 (1.71) ^b^	2.60 (1.61) ^a^	3.70 (1.90) ^b,c,d^
	Neutral	4.00 (1.54) ^d^	3.74 (1.50) ^b,c,d^	3.18 (1.46) ^a^	3.63 (1.45) ^b,c^	3.41 (1.64) ^b^	3.78 (1.51) ^c,d^
	Positive	3.88 (1.49) ^c,d^	4.04 (1.69) ^d^	4.48 (1.71) ^e^	4.05 (1.81) ^c,d,e^	4.09 (1.55) ^d,e^	4.41 (1.69) ^e^

The letters ^a–g^ indicate significantly different means on seven-point interval scales ranging from “Not at all” (=1) to “Very much” (=7).

**Table 3:** Intention to buy fruit juice with calcium claim [Mean (Standard Deviation)] by explicit and implicit HRMO and covariates.

		Explicit HRMO			Implicit HRMO		
		Low	Medium	High	Low	Medium	High
Gender	Male	3.12 (1.70) ^a^	3.67 (1.99) ^b^	3.90 (2.12) ^b^	3.31 (2.09) ^a^	3.30 (1.83) ^a^	3.96 (1.90) ^b,c^
	Female	3.47 (1.86) ^a,b^	3.83 (1.96) ^b^	3.84 (2.20) ^b^	3.63 (1.76) ^a,b^	3.41 (2.22) ^a^	4.03 (2.06) ^c^
Age group	18–25	3.32 (1.65) ^b,c^	4.22 (1.75) ^e^	4.12 (2.10) ^d,e^	3.66 (1.70) ^c,d^	3.53 (2.02) ^b,c,d^	4.20 (1.86) ^f^
	26–40	3.40 (1.48) ^b,c,d^	3.66 (1.93) ^c,d^	4.10 (2.08) ^d,e^	3.88 (2.13) ^c,d,e,f^	3.61 (2.25) ^b,c,d,e,f^	3.93 (1.76) ^d,e,f^
	41–50	3.71 (2.09) ^b,c,d,e^	3.71 (2.13) ^c,d,e^	4.06 (2.35) ^d,e^	3.31 (1.96) ^a,b,c^	3.73 (2.28) ^b,c,d,e,f^	4.26 (2.24) ^e,f^
	50+	3.00 (1.87) ^a,b^	3.29 (2.07) ^a,b,c^	2.84 (1.99) ^a^	3.10 (1.92) ^a,b^	2.65 (1.59) ^a^	3.44 (2.34) ^a,b,c,d,e^
Product familiarity	Non-user	2.45 (1.74) ^a^	2.62 (1.97) ^a^	2.46 (1.80) ^a^	2.48 (1.75) ^a,b^	2.20 (1.84) ^a^	2.73 (1.90) ^b^
	Light user	4.01 (1.50) ^b^	4.01 (1.62) ^b^	3.83 (1.85) ^b^	3.89 (1.68) ^c,d^	3.64 (1.74) ^c^	4.23 (1.60) ^d,e^
	Medium user	4.15 (1.06) ^b^	4.42 (1.65) ^b,c^	4.85 (1.94) ^c,d^	4.80 (1.27) ^e,f^	4.61 (2.35) ^c,d,e,f,g^	4.25 (1.49) ^d,e^
	Heavy user	3.88 (1.75) ^b^	5.20 (1.54) ^d^	5.62 (1.73) ^e^	4.69 (1.99) ^e,f,g^	5.06 (1.62) ^f,g^	5.37 (1.73) ^g^
General attitude towards functional foods	Negative	2.89 (1.70) ^a^	2.88 (2.02) ^a^	3.01 (2.02) ^a^	2.92 (1.86) ^b^	2.32 (1.73) ^a^	3.26 (1.93) ^b,c^
	Neutral	3.62 (1.85) ^c^	3.92 (1.91) ^c,d^	3.06 (1.87) ^a,b^	3.40 (1.83) ^c,d^	3.27 (1.97) ^b,c^	3.86 (1.90) ^d,e^
	Positive	3.58 (1.71) ^b,c^	4.14 (1.84) ^d^	4.65 (2.10) ^e^	4.13 (1.91) ^e,f^	4.04 (2.07) ^e^	4.56 (1.96) ^f ^

The letters ^a–g^ indicate significantly different means on seven-point interval scales ranging from “Not at all” (=1) to “Very likely” (=7).

### 3.3. Effects of Claim Type and HRMOs Linked to Calcium

**Figure 1:**
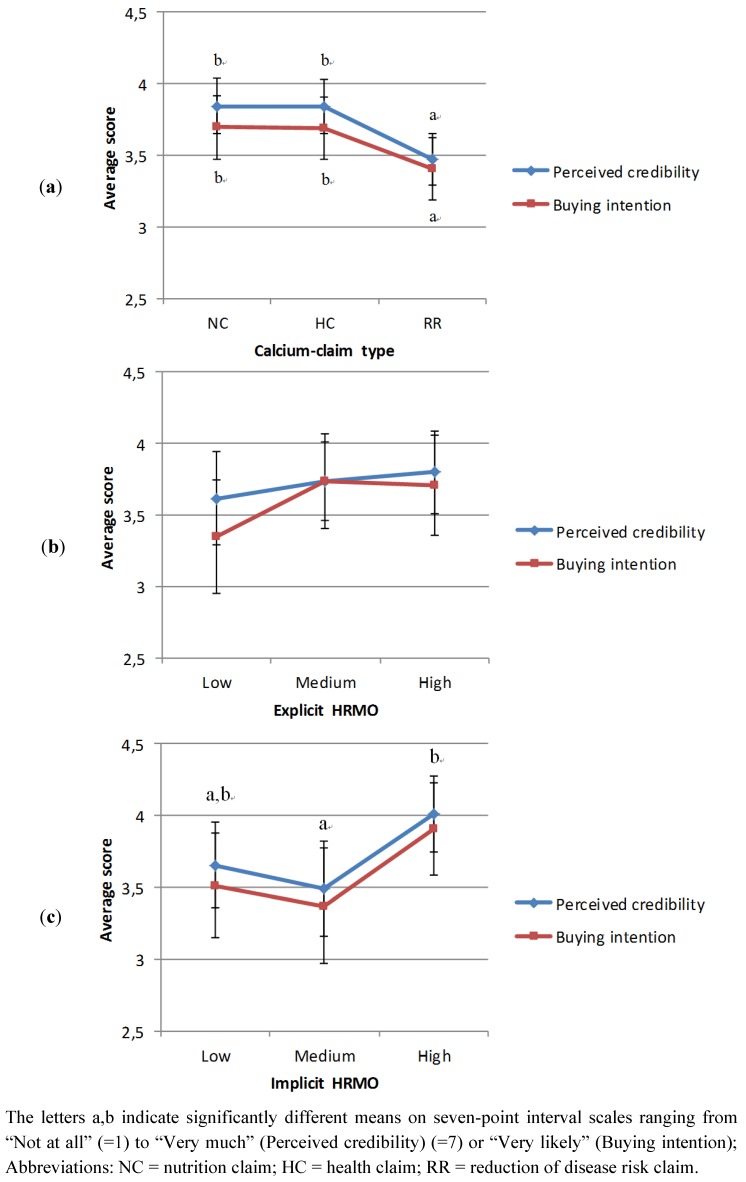
Main effects: estimated marginal means of perceived credibility and intention to buy as a function of (**a**) calcium-claim type, (**b**) explicit HRMO and (**c**) implicit HRMO.

Table 4 shows the results of the linear mixed models of perceived credibility and purchasing intention of calcium-enriched fruit juice with claims accounting for differences in product familiarity, socio-demographic and attitudinal characteristics. The effect of calcium-claim type was significant in both models (*p* < 0.001) and independent of participants’ explicit and implicit HRMOs, as indicated by the insignificant interaction effects (*p* > 0.05). Nutrition and health claims were perceived as more credible than the reduction of disease risk claim (Figure 1a). Participants were also more likely to buy calcium-enriched fruit juice with a nutrition claim or health claim than with a reduction of disease risk claim (Figure 1a). Although participants’ implicit HRMOs influenced their perceived credibility of the calcium-enriched fruit juice (Model 1, *p* = 0.045), it did not affect their purchasing intention of the product (Model 2, *p* = 0.068). The stronger the implicit HRMOs, the more credible the fruit juice was found to be (Figure 1b). No effect was observed for participants’ explicit HRMOs on the credibility and purchasing intention of the fruit juice (*p* > 0.05) (Figure 1c). 

**Table 4:** Estimates of fixed effects of claim type and HRMO on credibility of and intention to buy fruit juice with calcium claim.

Independent variables	Dependent variable			
	Credibility		Intention to buy	
	F	*p*-value	F	*p*-value
Claim type	25.372	<0.001	14.421	<0.001
Explicit HRMO	0.319	0.727	1.213	0.299
Implicit HRMO	3.188	0.043	2.457	0.087
Claim type × Explicit HRMO	1.525	0.194	0.855	0.491
Claim type × Implicit HRMO	0.779	0.539	1.228	0.299

## 4. Discussion

With the more stringent Regulation, aiming at a better understanding of health claims by the average consumer, there is a need for more insights into consumers’ understanding of and reactions to the claimed health benefits and their relevance. While there is a growing body of literature on consumers’ perception of food claims, to the authors’ knowledge, only two studies examined motivational factors underpinning consumers’ reactions to claims [14,15]. Specifically, this study reports on the effects of consumers’ HRMOs together with the calcium-claim type (nutrition claim, health claim and reduction of disease risk claim) on their perceived credibility and purchasing intention of calcium-enriched fruit juice. 

A distinction was made between explicit and implicit HRMOs to obtain a more complete understanding of the motivational underpinnings of the health-related behavior [38]. This multidimensional perspective on the meaning of health has been successful in identifying target health-related motive segments [17,18]. While previous studies have focused primarily on the effects of explicit motives on health-related behavior [39,40,41], evidence suggests the importance of implicit motives in predicting health-related behaviors [19]. The present study confirmed the relative importance of implicit compared with explicit HRMOs in determining perceived credibility of calcium-enriched fruit juice in a sample of Belgian consumers. However, in terms of consumers’ purchasing intention, both the effects of explicit and implicit HRMOs were not significant. This finding suggests that strong implicit HRMOs are not sufficient for consumers to intend purchasing functional foods despite positive evaluations in terms of product credibility. The gap between the perceptual evaluative measures and behavioral intention were previously also observed by Verbeke *et al.* [28]. Moreover, consumers’ credibility and claimed intention may not necessarily translate into choice and dietary behavior. 

Overall, the claim type was found to be a more important determinant of consumers’ perceived credibility than their health-related motive orientation. Many studies have shown differences in claim perceptions depending on the combinations of carrier product, functional ingredient and type of claim [20,29,32,42,43]. In general, health claims are preferred above reduction of disease risk claims. Additionally, in this study, the effect of claim type was found to be independent of participants’ explicit and implicit HRMOs, as indicated by the insignificant interaction effects. 

Practical implications from this study mainly pertain to the formulation and communication of health claims, given that the explicit HRMOs more or less refer to the currently claimed health benefits. According to our findings for the specific case of calcium-enriched fruit juice, health claim formulations and communications that refer to the implicit values of calcium for personal health are expected to be more effective, especially among younger consumers, who are already familiar with the product and have more favorable attitudes towards functional foods. Since health claims can only improve food choices if consumers both understand the health benefit and find it relevant for their personal health, future research is needed on whether consumers understand the content of the claims and whether this understanding is associated with its perceived relevance.

Some limitations should be acknowledged when interpreting our findings. A first limitation pertains to the choice for the specific product-ingredient-claim combination. A comparison between different products varying in perceived healthiness and degree of familiarity would be interesting, since diverse products may elicit different responses to nutrition [44,45]. For example, some foods may rather be chosen for satisfying a predominant health motive, whereas others may rather be chosen to satisfy hedonic motives. This study focused only on the presence of a qualifying nutrient (*i.e.*, calcium), without addressing the potential absence of a disqualifying nutrient (e.g., added sugar). Since the level of perceived importance is higher for qualifying than for disqualifying nutrients, different reactions to claims could be expected with other nutrients of investigation [46]. Although fruit juices have a healthy image [23] and calcium is a well-known positive or qualifying nutrient [45], the less natural combination of juice with calcium was previously found to result in skepticism and lower evaluations of the product-ingredient-claim combination [28,47,48,49]. Furthermore, the study was limited to the use of a single example formulation to represent each of the claims. Future studies are recommended to investigate different wordings or visual representations of the claims, *i.e.*, claim presentation format or label format effects [22,50,51]. 

Second, the specificity of the study sample does not allow generalization to specific defined populations. Future studies are recommended to investigate whether the findings of this study hold equally among particular consumer segments, e.g., people with a specific interest in the product, because of health reasons or consumers who have a family history of food-related lifestyle or other diseases, such as cardiovascular disease [52] or osteoporosis, or in other countries with a different history of use of food claims. 

Third, the present study, just like most consumer research, depended on self-reported data. Although studies using self-reported behavior provide valuable insights, they likely suffer from so-called social desirability bias and, hence, may deviate from actual behavior [53]. Therefore, more experimental and observational research is recommended using real product settings and shopping environments. As such, potential interactions between brand name, corporate image and perception of claims can be investigated. 

## 5. Conclusions

This study showed the relative importance of implicit compared to explicit motive orientations in explaining consumers’ reactions to a calcium-enriched food with claim. The implicit meaning and relevance attached to health benefits from calcium emerged as more predictive for consumers’ appeal of claims on calcium-enriched fruit juice than the explicit meaning and value of calcium for health. More important than consumers’ HRMOs is the effect of claim type on the perceived credibility and purchasing intention of the product, where health claims were most favored. Based on the presented findings, health claim communications that refer to the implicit values of calcium for personal health are expected to be more effective, especially among younger consumers, who are more familiar with the product and have more favorable attitudes towards functional foods. Since health claims may only improve food choices if consumers understand the relevance of the health benefits and the claimed health effects, it is recommended to further investigate the motivational factors in combination with consumers’ understanding of the claims. 
